# 2021 - State of our JCMR

**DOI:** 10.1186/s12968-021-00840-2

**Published:** 2022-03-04

**Authors:** Warren J. Manning

**Affiliations:** 1 Departments of Medicine (Cardiovascular Division) and Radiology, Beth Israel Deaconess Medical Center, Harvard Medical School, Boston, Massachusetts 02215 USA; 2 JCMR Editorial Office, Boston, Massachusetts 02215 USA

## Abstract

There were 89 articles published in the *Journal of Cardiovascular Magnetic Resonance* (*JCMR*) in 2020, including 71 original research papers, 5 technical notes, 6 reviews, 4 Society for Cardiovascular Magnetic Resonance (SCMR) position papers/guidelines/protocols and 3 corrections. The volume was up 12.7% from 2019 (n = 79) with a corresponding 17.9% increase in manuscript submissions from 369 to 435. This led to a slight increase in the acceptance rate from 22 to 23%. The quality of the submissions continues to be high. The 2020 JCMR Impact Factor (which is published in June 2020) slightly increased from 5.361 to 5.364 placing us in the top quartile of Society and cardiac imaging journals. Our 5 year impact factor increased from 5.18 to 6.52. Fourteen years ago, the *JCMR* was at the forefront of medical and medical society journal migration to the Open-Access format. The Open-Access system has dramatically increased the availability and citation of *JCMR* publications with accesses now exceeding 1.2 M! It takes a village to run a journal. *JCMR* is blessed to have a group of very dedicated Associate Editors, Guest Editors, Journal Club Editors, and Reviewers. I thank each of them for their efforts to ensure that the review process occurs in a timely and responsible manner. These efforts have allowed the *JCMR* to continue as the premier journal of our field. My role, and the entire process would not be possible without the dedication and efforts of our new managing editor, Jennifer Rodriguez, whose premier organizational efforts have allowed for streamlining of the review process and marked improvement in our time-to-decision (see later). As I begin my 6th and final year as your editor-in-chief, I thank you for entrusting me with the *JCMR* editorship*.* I hope that you will continue to send us your very best, high quality manuscripts for *JCMR* consideration and that our readers will continue to look to *JCMR* for the very best/state-of-the-art CMR publications. The editorial process continues to be a tremendously fulfilling experience and the opportunity to review manuscripts that reflect the best in our field remains a great joy and true highlight of my week!

## Background

In accordance with Open-Access publishing guidelines of our publisher, BMC, the *Journal of Cardiovascular Magnetic Resonance (JCMR)* articles are published on-line in a continuous fashion and in chronologic number order, with no collating of the articles into sections or special thematic issues. In addition, due to the variability in the author galley review process, articles are sometimes not listed in chronologic order on the *JCMR* web site. As a result, our second editor-in-chief, Dr. Dudley Pennell initiated an annual review of all *JCMR* publications into broad areas of interest or themes, allowing readers to view areas of interest in a single article in relation to each other and contemporaneous *JCMR* publications. Though I believe this review would be a valuable asset for our readership, I have chosen not to continue this tradition so as to decrease our self-citation rate and potential “delisting” from the annual Impact Factor calculation. Instead, similar to last year, I will focus on conveying information regarding the editorial process as a “State of our *JCMR*” summary.

The *JCMR* is the official publication of the Society for Cardiovascular Magnetic Resonance (SCMR). The *JCMR* published 89 articles in 2020, including 71 original research papers, 5 technical notes, 6 reviews, 4 SCMR position papers/guidelines/protocols and 3 corrections. The 2020 publication volume was up 12.7% from 2019 (n = 79) with a corresponding 17.9% increase in manuscript submissions from 369 to 435. This led to a slight increase in the acceptance rate from 22 to 23% (the slight mathematical difference in acceptance/submissions is related to submission year and publication year).

In July 2018, the article processing charge (APC) structure changed with manuscripts for which an SCMR member is  the first, co-first, senior or corresponding author receive an 80% discount to the full $2500 APC. Reduced APC fees are also available to those from BMC membership institutions, submitting authors from lower income countries, and for those who request a waiver due to financial hardship. APCs are waived for invited reviews and for Society publications. The APC will not be changing for 2022.

**Fig. 1:**
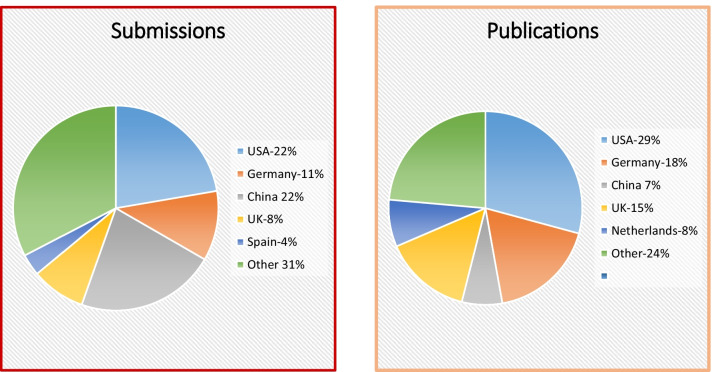
**A** Pie chart of 2020 *JCMR* submissions *submissions* by country. **B** Pie chart of 2020 *JCMR* *publications* by country

For 2020, the United States (n = 97) and China (n = 96) were the source of nearly 50% of all *JCMR* publications followed by Germany (n = 48) and the United Kingdom (n = 37) (Fig. 1). The top three countries for publications were the United States (n = 26), Germany (n = 16) and the United Kingdom (n = 13) (Fig. 1).

### Impact factor

Though only one of many journal metrics and not a consideration in our review process, the Clarivate Impact Factor is nonetheless a well-known metric with which many readers are familiar and is a metric often considered by both authors and readers. I am pleased to report that the 2020 *JCMR* Impact Factor (which was released in June 2021 and is based on manuscripts published in 2018 and 2019 that were cited in 2020), slightly increased from 5.361 to 5.364. This impact factor means that the *JCMR* papers that were published in 2018 and 2019 were cited on average approximately 5.4 times in 2020. This puts *JCMR* well positioned in the top tertile (37/142) of journals in the broad categories of “Cardiac and Cardiovascular Systems” and the top quintile (20/133) of “Radiology, Nuclear Medicine and Medical Imaging.” In addition, our Journal Citation Indication is 1.44, meaning JCMR has 44% greater citation impact than the average in its category! The 2021 *JCMR* impact factor will be released in June 2022, and I will keep you abreast of the results (follow us on Twitter!).

Perhaps more important than the Impact Factor is the frequency that *JCMR* articles are accessed. Our open-access format allows for much greater visibility for our authors with the 2020 *JCMR* annual digital access now exceeding 1,200,000!!—a threshold not achievable with a subscription/print publication of a Society journal. Open-access has “leveled the playing field” so that an electronic search allows *JCMR* manuscripts to rise to awareness and to then be downloaded without cost. This is a great benefit to our readers, to the greater scientific community, and to our authors.

## *JCMR* editor-in-chief leadership

**Fig. 2:**
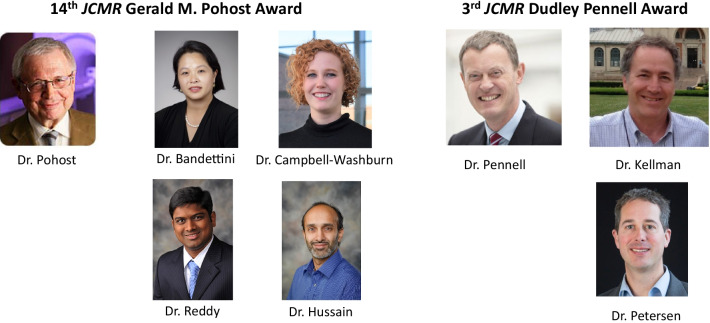
2020 JCMR 14th Gerald M. Pohost Awardee/runner up. 3rd Dudley Pennell Awardee/runner-up

Dr. Gerald Pohost (Fig. 2) from the University of Alabama at Birmingham and University of Southern California, Los Angeles, California, USA was the *JCMR* inaugural editor-in-chief. In 2006, he was succeeded by Professor Dudley Pennell (Fig. 2) of the Royal Brompton Hospital, London, England. Since December 2016, the *JCMR* editorial office has been located at the Beth Israel Deaconess Medical Center, Boston, Massachusetts, USA under my leadership. My 6 year term will end at the end on December 31, 2022, and I have decided not to request a second term. My decision was based on numerous personal and professional issues and does not reflect any decline in my passion for CMR or the *JCMR*. The *JCMR* associate editors, journal club editors, and SCMR leadership have been aware of my decision for several months—with plans underway to identify my successor. I anticipate a new *JCMR* editor-in-chief will be selected by the SCMR leadership in the coming months and our Boston team will work towards a smooth transition at the end of 2022 with closure of the Boston office in April 2023. If you are interested in the editor-in-chief role, I suggest that you contact SCMR leadership!

## 2020* JCMR* editorial and management team

The *JCMR* Associate Editors (Table 1) reflect the international and diverse spectrum of the SCMR. This past year, Dr. Tim Leiner returned to the associate editorial board after stepping away during his term as president of the International Society of Magnetic Resonance in Medicine (ISMRM). It is great to have Tim’s wisdom back at our weekly editorial board meetings. Drs. Yuchi Han and Tim Leiner didn’t miss a beat of their *JCMR* associate editorial participation despite their professional moves this year to Ohio State University and the Mayo Clinic, respectively. Our other Associate Editors include Drs. Rene Botnar (UK/Chile), John Greenwood (UK), Dara Kraichman (USA), Robert Lederman (USA), Reza Nezafat (USA), Amit Patel (USA), Joshua Robinson (USA) and Connie Tsao (USA). Dr. Long Ngo (USA) continues to serve as our statistical editor. Drs. Juan Lopez-Mattei (USA) and Purvi Parwani (USA) are busy every week disseminating *JCMR* news as our Social Media/Twitter editors.

**Table 1:** JCMR Associate Editors, Statistical Editor, Journal Club Editors, and Social Media Editors

*Associate Editors*	
Rene Botnar	King’s College, London, UK/Chile
John Greenwood	University of Leeds, UK
Yuchi Han	Ohio State University, USA
Dara Kraichman	Johns Hopkins University School of Medicine, USA
Robert Ledeerman	National Institutes of Heart, Lung, and Blood Institute, USA
Tim Leiner	Mayo Clinic, USA
Reza Nezafat	Beth Israel Deaconess Medical Center, USA
Amit Patel	University of Virginia, USA
Joshua Robinson	Northwestern University, USA
Connie Tsao	Beth Israel Deaconess Medical Center, USA
*Statistical Editor*	
Long Ngo	Beth Israel Deaconess Medical Center, USA
*Journal Club Editors*	
Scott Flamm	Cleveland Clinic, USA
Raymond Kwong	Brigham and Women’s Hospital, USA
Matthias Stuber	University of Lausanne, Switzerland
*Social Media Editors*	
Juan Lopez-Mattei	Lee Health Heart, USA
Purvi Parwani	Loma Linda University Health, USA

**Fig. 3:**
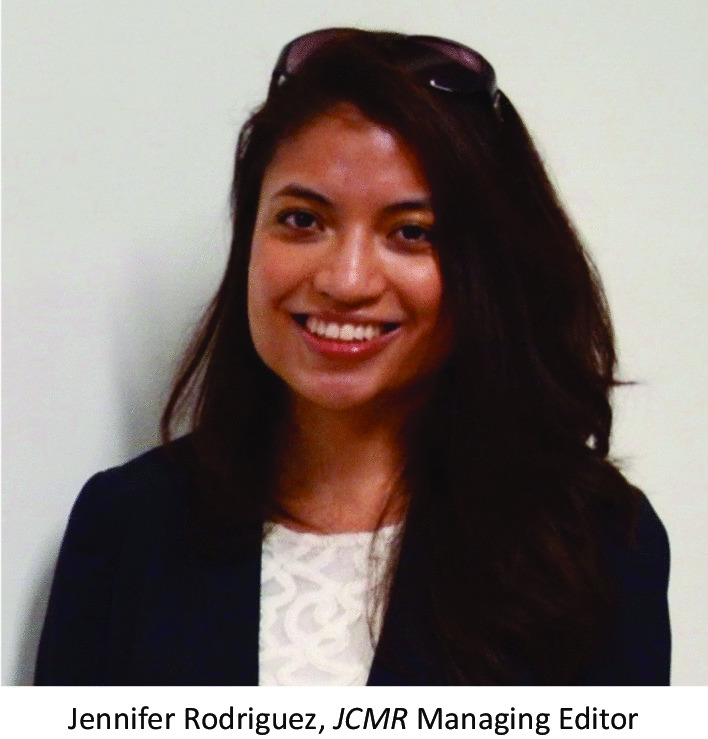
Jennifer Rodriguez is the *JCMR* managing editor

Jennifer Rodriguez (jcmroffice@scmr.org) joined the *JCMR* managing staff as an assistant managing editor in mid-2019 and was promoted to managing editor in January 2021 (Fig. 3). Jennifer has made tremendous progress in keeping me and the entire manuscript review process organized and on schedule. As a result, we have seen a marked decrease in our time to first decision time from a mean of 63 days in 2019 to 36 days for the first 6 months of 2021! I hope our authors have felt this tangible difference.

## 2020* JCMR* Journal Club

A highlight of 2020 was the introduction of our *JCMR* Journal Club just as the COVID-19 pandemic was bursting across the globe in March 2020. These monthly one-hour webinars are held on the 2nd Wednesday of the month at 11am ET. A link for the monthly registration is on the JCMR (https://jcmr-online.biomedcentral.com/) and SCMR (www.scmr.org) websites. These monthly *JCMR* Journal Clubs are hosted by one of our three Journal Club Editors, Drs. Scott Flamm (clinical), Raymond Kwong (clinical) and Matthias Stuber (non-clinical). On a rotating basis, each editor choses a manuscript that was recently published in *JCMR* After a brief Journal Club Editor introduction of the topic, the presenting author gives a 25–30 min presentation followed by a spirited 30 min discussion. We continue to offer CME for reading the manuscript and hope to provide CME for Journal Club attendance in the coming year. Please join > 100 of your colleagues every month for an informative presentation and discussion! Don’t worry if you missed one. Recordings of the monthly webinars and a CME link are provided on the SCMR web site. Check them out!

Like other JCMR activities, the *JCMR* Journal Club is a village effort. In addition to our 3 talented Journal Club editors, I very much appreciate the strong administrative assistance of Lauren Small from the SCMR managing office who was succeeded by Sarah Mania this past July.  Lauren and now Sarah are responsible for coordinating registration, the speaker presentations, CME, Zoom operations and recording, and subsequent posting of the monthly *JCMR* Journal Club recording on the SCMR website. The 2020 JCMR Journal Club selections were on a wide variety of topics (Table 2).

**Table 2:** 2020 Monthly JCMR Journal Club Editor, Presenter, Manuscript

Date	Editor	Presenter	Manuscript
3/10/2020	Raymond Kwong	Mohammad Alkhalil	Hyper-acute cardiovascular magnetic resonance T1 mapping predicts infarct characteristics in patients with ST elevation myocardial infarction. [20]
4/15/2020	Scott Flamm	Thu-Thao Le	The application of exercise stress cardiovascular magnetic resonance in patients with suspected dilated cardiomyopathy. [22]
5/13/2020	Raymond Kwong	Haikun Qi	Respiratory motion-compensated high-resolution 3D whole-herat T1p mapping. [23]
6/10/2020	Scott Flamm	G.J.H. Snel	Cardiovascular magnetic resonance native T2 and T2* quantitative values for cardiomyopathies and heart transplantations: a systematic review and meta-analysis. [5]
7/8/2020	Matthias Stuber	Adrienne E. Campbell-Washburn	A comparison of cine CMR imaging at 0.55 T and 1.5 T. [15]
8/12/2020	Raymond Kwong	Eike Nagel	Sub-segmental quantification of single (stress)-pass perfusion CMR improves the diagnostic accuracy for detection of obstructive coronary artery disease. [27]
9/16/2020	Scott Flamm	B. Domenech-Ximenos	Prevalence and pattern of cardiovascular magnetic resonance late gadolinium enhancement in highly trained endurance athletes. [28]
10/14/2020	Raymond Kwong	Amol S. Pednekar	Breath-hold and free-breathing quantitative assessment of biventricular volume and function using compressed SENSE: a clinical validation in children and young adults. [32]
11/11/2020	Matthias Stuber	Thomas P. Craven	Exercise cardiovascular magnetic resonance development, current utility, and future applications. [3]
12/8/2020	Scott Flamm	Teresa Correia and Claudia Prieto	Accelerated high-resolution free-breathing 3D whole-heart T2-prepared black-blood and bright-blood CMR. [33]

Continuing medical education (CME) is offered for reading of the manuscript and is a complimentary benefit for SCMR members

## Manuscript review process, omissions, and suggestions

I reviewed the manuscript submission process in my report last year [1] and will further expand on this below.

All manuscripts are submitted and processed through the http://www.jcmr-online.org website. I encourage all authors to closely follow the guidelines so as not to delay the review process. By far, the most error that leads to review delay is the omission of the names and contact information for at least two suggested reviewers in the cover letter. I ask authors to use *JCMR* preferred abbreviations (https://jcmr-online.biomedcentral.com/submission-guidelines/preparing-your-manuscript/abbreviations) and to use the terms “CMR” and “cardiovascular magnetic resonance” rather than “cardiac magnetic resonance.” While the abbreviation issue does not delay the review, it adds additional burden to the prepublication editing process.

I encourage authors to carefully consider the number of significant digits and reported p values in their manuscripts. For example, when reporting native T1 and standard deviation, would report to the nearest ms and not to the X.X ms or X.XX ms. While technically accurate, reporting T1 to this level of accuracy has no clinical relevance. Similarly, when reporting p values for the sample sizes of most *JCMR* publications, a value of < 0.001 is a reasonable limit.

*JCMR* subscribes to the BMC guidelines for manuscript citations (https://www.biomedcentral.com/getpublished/editorial-policies#citations) which I will summarize below.

Research articles and non-research articles (e.g. Reviews and Position Papers) should cite appropriate and relevant literature in support of the claims made. Excessive self-citation, coordinated efforts among authors to collectively self-cite, gratuitous and unnecessary article citation or any other form of citation manipulation are inappropriate and strongly discouraged.

Authors should consider the following guidelines when preparing their manuscript:

- Statements that rely on external sources of information (i.e. not the authors' own new ideas or findings or general knowledge) should use a citation.
- Authors should cite the original work rather than a review article that cites an original work.
- Authors should ensure that their citations are accurate.
- Authors should not cite sources that they have not read.
- Authors should cite the most appropriate publication and not preferentially cite their own or their friends’, peers’, or institution’s publications.
- Authors should avoid citing work solely from one country.
- Authors should not use an excessive number of citations to support one point.
- When possible, authors should prioritize peer-reviewed sources and should not cite advertisements or advertorial material.

After manuscript submission and BMC office confirmation that the manuscript is in the appropriate format (abstract, text, references, figures, tables, supplements), the manuscript is sent to the Boston office for initial review. Within 48 business hours, I assess the manuscript for its appropriateness for the *JCMR* readership and its overall likely priority for publication. Approximately 5% of submitted manuscripts are deemed inappropriate for the *JCMR* audience (non-CMR topic) or very unlikely to reach sufficient priority for acceptance (e.g., case reports/very small case series, unsolicited reviews). These manuscripts are quickly returned to the author(s) so as to expedite submission to a more appropriate journal. If appropriate, the authors are offered the opportunity to directly forward their manuscript to another BMC open-access publication.

For manuscripts deemed appropriate for consideration, an associate editor is assigned and reviewer assignments are then requested. Manuscript evaluations are simultaneously requested from up to 5 reviewers (with special consideration for the 2 author suggested reviewers) until confirmed acceptance has been received by 3 reviewers. Reviewers are asked to follow a specific format and to return their review within 2 weeks of acceptance. We are fortunate to have over 900 reviewers (but are always looking to expand our reviewer pool and encourage all members/innovators/leaders of the CMR field to apply to be a reviewer. If you are interested in becoming a *JCMR* reviewer, please contact our managing editor, Jennifer Rodriguez at jcmroffice@scmr.org*.*

When at least two (of 3 agreed) reviews have been received by noon Friday, the manuscript is scheduled for discussion at our weekly associate editorial board meeting held every Tuesday from 9:30 to 10:30 a.m. ET. When I am out of town/unavailable, the associate editors continue to meet so as to not delay the publication process. At each meeting, 4–12 manuscripts may be discussed. The manuscript decisions at that meeting include.

1. *Accept* (most commonly after one or more major and/or minor revisions)
2. *Minor revision*—No new experiments are requested, relatively minor text changes or analyses are requested; 30 day turn-around with submission of clean and track-change versions of the manuscripts along with point-by-point response(s) to the reviewers/editors. These manuscripts are generally *not* returned to the reviewers for their assessment. We expect > 98% acceptance.
3. *Major revision*—Substantial text and/or analyses are needed. This may include additional experiments; 90 day turn-around with submission of clean and track-change versions of the manuscripts along with point-by-point responses to the reviewers/editors. These manuscripts are sent back to the original reviewers to confirm that their concerns have largely been addressed. Overall, ~ 60% ultimate acceptance is anticipated
4. *Denovo resubmission*—Substantial new experiments/analyses are needed or change in manuscript focus; unlimited turn-around time with submission of clean and track-change versions of the manuscripts along with point-by-point responses to the reviewers/editors. These manuscripts are usually sent to the original reviewers to confirm their concerns have been addressed. Overall, we anticipate ~ 40% acceptance.
5. *Decline* Authors are offered the opportunity to have their manuscript considered by another journal in the BMC family with inclusion of the *JCMR* reviews to expedite the process.

When a manuscript is accepted, I then edit the submission to be consistent with *JCMR* style/abbreviations (see https://jcmr-online.biomedcentral.com/submission-guidelines/preparing-your-manuscript/abbreviations) before final submission to BMC for galley production. The galleys are first sent to the corresponding author and finally to me for final sign-off. I then identify a postage stamp (remember those!) image for publication in *JCMR* web site listing and to accompany the @JournalofCMR twitter feed. The manuscript is usually published on-line within a week of my final sign-off. Along with BMC, Drs. Juan Lopez-Mattei and Purvi Parwani handle the social media dissemination of the manuscript’s publication.

Upon taking my position as editor-in-chief in 2017, my goal was to have first decision within 40 days of receipt for 60% of manuscripts, a process that is dependent on timely return of reviews. I am pleased to report that we have more than achieved this goal for 2021—thanks in large part to the strong efforts of our new managing editor, Jennifer Rodriguez. Delays are due to multiple issues—if the two reviews markedly differ in their assessment/recommendation (~ 25% of the time) or the associate editor feels we need additional information; we may then delay a decision until the third review has been received or solicit a fourth. We may also to seek the counsel of our statistical reviewer, Dr. Long Ngo. If any of these occur, we try to alert the corresponding author.

We recognize that the review process is not perfect. We may not have sent the manuscript to the best reviewers (your suggestions help, or the best reviewers may have declined our invitation). The reviewer may have misinterpreted the manuscript (we try to catch this at our weekly associate editorial meeting).

Sometimes you will find the editorial decision is different from your perception of the review(s). This is because we do our best to objectively assess the science, presentation, and appropriateness for the *JCMR* audience. The review(s) help, but we also ask ourselves these four questions:

1. Is the study scientifically sound?
2. Are the Methods, Results, and Discussion appropriately presented?
3. Is the work novel? Does the study extend or clarify our current understanding or is it a confirmation of a prior report?
4. Will our readership be interested or informed by the topic?

Anonymized reviews are returned to the authors. We continue to work with BMC so that we can post anonymized reviews for published manuscripts on the *JCMR* web site. In contrast to some open-access journals, I do not anticipate posting of submitted (but not accepted) manuscripts or inclusion of prior versions of an accepted manuscript as I am concerned such postings will be confusing to the reader. Stay tuned as we hopefully bring you anonymized reviews in 2022.

## All manuscripts submitted to the journal cannot be under simultaneous consideration by another journal

All work submitted to the *JCMR* must be original and cannot be under consideration by another journal until a decision is made by the* JCMR*. Fortunately, this past year we have not had any known occurrence of simultaneous submissions; but have unfortunately become aware of this issue in the past. When this does occur, the manuscript is immediately withdrawn from further consideration and the authors are put on administrative warning.

## Reviewer recognition—Gold Star Reviewers

Reviewers are a key component to the success of the *JCMR*. As a recognition of reviewers, at the 2021 SCMR Annual meeting we recognized our 135 “Gold Star” Reviewers for 2020 (Table 3). Gold Star reviewers are those individuals who reviewed at least 3 *JCMR* manuscripts in 2020, with reviews both of high quality and timeliness. Please join the ranks of *JCMR* reviewers and strive to be a Gold Star reviewer! As an added incentive, reviewers have the option to receive continuing medical education (CME) credit for providing a review.

**Table 3:** 2020 JCMR Gold Star Reviewers listed in alphabetical order by last name.

Ganesh Adluru
Bradley D Allen
Tarek Alsaied
Ashish Aneja
Janine Arruda
Daniel Auger
Ryan Avery
Adrianus J. Bakermans
W. Patricia Bandettini
Anna Baritussio
Tamer Basha
Meinrad Beer
Vasili Berdoukas
David Alan Bluemke
Paco Bravo
Christopher Bruce
Claudia Calcagno
Joseph Camarda
Andrea Cardona
YuCheng Chen
Michael Chuang
Taylor Chung
Francisco Contijoch
Bram F Coolen
Erica Dall'Armellina
Ranil De Silva
Lajja Desai
Robert R. Edelman
Michael Elliott
Emil Knut Stenersen Espe
Ahmed Fahmy
Domenico Filomena
Marianna Fontana
Sunil J. Ghelani
Nilesh R Ghugre
Vasu D. Gooty
Lindsay Griffin
Lars Grosse-Wortmann
Heynric B. Grotenhuis
Ying kun Guo
Hassan Haji-Valizadeh
Mehdi Hedjazi Moghari
Markus Henningsson
Lazaro Eduardo Hernandez
Aaron Hess
Peng Hu
Edward Hulten
Tevfik F Ismail
Jason Nathaniel Johnson
Jennifer Jordan
Shingo Kato
Keigo Kawaji
Won Yong Kim
Gert Klug
Grigorios Korosoglou
Sebastian Kozerke
Ramkumar Krishnamurty
Selcuk Kucukseymen
Deborah Kwon
Seung-Pyo Lee
Zhitao Li
Timo Liimatainen
Joao A.C. Lima
Harold Litt
Massimo Lombardi
Jimmy Lu
Minjie Lu
Julian Luetkens
Pierre-Yves Marie
Michael Markl
Shiraz Maskatia
Anthony MerloccoLorenzo Monti
Vivek Muthurangu
Shintaro Nakano
Thomas Neuberger
Christopher Nguyen
TD Nguyen
Laura Olivieri
Declan O'Regan
Ellen Ostenfeld
Paras Parikh
Dana Peters
Steffen E Petersen
Prashob Porayette
Valentina O Puntmann
Francesca Raimondi
Shams Rashid
Nathaniel Reichek
Pierangelo Renella
Jose F Rodriguez Palomares
Idan Roifman
Frederick Ruberg
Tobias Rutz
Sudip Saha
Hajime Sakuma
Michael Schär
Ehud Schmidt
Andrew David Scott
Aurelio Secinaro
Dipan J. Shah
Moneal Shah
Sujata M Shanbhag
Gaurav Sharma
Chetan Shenoy
Timothy Slesnick
Sahar Soleimani
Monvadi Barbara Srichai-Parsia
Jordan B. Strom
Matthias Stuber
Avan Suinesiaputra
Qian Tao
Michael D. Taylor
Connie Tsao
Robert Tunks
Pim van Ooij
Akos Varga-Szemes
Miguel Silva Vieira
Inga Voges
Maximilian von Roeder
Ralf Wassmuth
Sebastian Weingärtner
David Wendell
Jos J Westenberg
John Whitaker
Timothy C Wong
Pamela Woodard
Katherine Wu
Yibin Xie
Hsin-Jung Yang
Yang Yang
Alistair Young
Chun Yuan
Karolina Zareba
Chengcheng Zhu

## Conflict-of-interest, reviews, SCMR guideline/position manuscripts and SCMR Committee papers

Conflict-of-interest manuscripts, those for which a member of the associate editorial board is either an author or closely associated with an author, are independently handled by a Guest Editor (Table 4) chosen by me. Neither I nor any of the associate editorial board are involved with reviewer selection or with manuscript decision. Our managing editorial office assists the Guest Editor with the administrative software/Editorial Manager. If a conflict-of-interest manuscript is accepted, the Guest Editor is recognized in the *JCMR* publication with the text “Dr. XX served as a *JCMR* Guest Editor for this manuscript.”

**Table 4:** 2020 JCMR Guest Editors

Andrew E. Arai	USA
Hugh Calkins	Johns Hopkins University, USA
Raymond Chan	University of Toronto, Canada
Paul Finn	University of California at Los Angeles, USA
Matthias Gero Friedrich	McGill University, Canada
Robert Judd	Duke University, USA
Tim Leiner	Utrecht University, The Netherlands
Debiao Li	Cedars-Sinai Medical Center, USA
Daniel R. Messroghli	Charité-University Medicine Berlin, Germany
John Oshinski	Emory University, USA
Ellen Ostenfeld	Lund University, Sweden
Nathaniel Reichek	Stony Brook University, USA
Matthias Stuber	University of Lausanne, Switzerland
Robert Weiss	Johns Hopkins University, USA

The *JCMR* does not accept unsolicited reviews. Authors are encouraged to contact me before submitting any reviews. In general, reviews are authored by individuals considered experts in the field and receive considerable attention/downloads. All solicited reviews follow the usual peer-review process. Several reviews were published in 2020 (Table 5), including reviews on normative values [2] exercise CMR [3], native T2 and T2* for cardiomyopathies and heart transplantation [4, 5], the role of CMR in women [6], and highlights of the 2020 23^rd^ SCMR Scientific Session [7].

**Table 5:** 2020 JCMR Reviews and SCMR Position Statements

First author	Publication
*2020 JCMR reviews*	
Craven et al.	Exercise cardiovascular magnetic resonance: development, current utility and future applications.* [3]
Snel GJH et al.	Cardiovascular magnetic resonance native T2 and T2* quantitative values for cardiomyopathies and heart transplantations: a systematic review and meta-analysis. [4, 5]
Bucciarelli-Ducci, et al.	Cardiovascular disease in women: insights from magnetic resonance imaging. [6]
Grosse-Wortman, et al.	Highlights of the 2020 23rd Society for Cardiovascular Magnetic Resonance Scientific Sessions. [7]
Kawel-Boehm et al.	Reference ranges ("normal values") for cardiovascular magnetic resonance (CMR) in adults and children: 2020 update. [2]
*2020 SCMR position papers*	
Schulz-Menger et al.	Standardized image interpretation and post-processing in cardiovascular magnetic resonance—2020 update: Society for Cardiovascular Magnetic Resonance (SCMR): Board of Trustees Task Force on Standardized Post-Processing. [8]
Kramer et al.	Standardized cardiovascular magnetic resonance imaging (CMR) protocols: 2020 update. [9]
Leiner et al.	SCMR Position Paper (2020) on clinical indications for cardiovascular magnetic resonance. [10]
Allen et al.	Society for Cardiovascular Magnetic Resonance (SCMR) guidance for re-activation of cardiovascular magnetic resonance practice after peak phase of the COVID-19 pandemic. [12]
Han et al.	Society for Cardiovascular Magnetic Resonance (SCMR) guidance for the practice of cardiovascular magnetic resonance during the COVID-19 pandemic. [11]

^*^CME offered for this manuscript

The *JCMR* is the official publication of the SCMR. As such, SCMR Guidelines and Position papers endorsed by the Full (or Executive) SCMR Board(s) do *not* undergo peer review. I review these manuscripts for consistency with *JCMR* style and abbreviations. They are then published in an expeditious manner. In addition to Society Position papers on standardized image interpretation and post-process [8], updated standardized CMR protocols [9], and clinical indications for CMR [10], we published several Covid-specific SCMR position papers in 2020 [11, 12] (Table 5). I have encouraged Society leadership to have updates of the Guideline papers to be on a 5 year renewal cycle, with rotation in authorship as well. In contrast to SCMR Guidelines and Position papers, SCMR Committee approved manuscripts undergo the usual *JCMR* peer review process albeit with an anticipation that they will ultimately be published in the *JCMR.* SCMR Committee publications in 2020 are listed in Table 5.

## SCMR case of the week series

While the *JCMR* does not accept case reports, for many years, the SCMR web site has an active “Case of the Week” (https://scmr.org/page/caseoftheweekLDGPG) series, currently coordinated by Dr. Sylvia Chen. In 2021, we published the 2019 Case series as a single manuscript [13]. We plan to make this unified publication an annual occurrence in *JCMR* to allow for these illustrative cases to be more widely available to search engines.

## Continuing Medical Education (CME) *JCMR* Journal Club

For over 4 years we have been offering on-line CME credit for the benefit of our clinician readers and is a free benefit for SCMR members—allowing them to more easily fulfill the CME criteria for maintenance of their Level II or III certification [14]. This program has been a great success and was greatly expanded with 20 manuscripts in 2020 alone! (Table 6). Please see http://scmr.peachnewmedia.com/store/provider/custompage.php?pageid=20 for the complete listing.

**Table 6:** 2020 JCMR manuscripts chosen for continuing medical education (CME)

Dreisbach et al.	Cardiovascular magnetic resonance based diagnosis of left ventricular non-compaction cardiomyopathy: Impact of cine bSSFP strain analysis. [19]
Alkhalil et al.	**Hyper-acute cardiovascular magnetic resonance T1 mapping predicts infarct characteristics in patients with ST elevation myocardial infarction.** [20]
Bandettini et al.	**A comparison of cine CMR imaging at 0.55 T and 1.5 T.** [15]
Kim et al.	Myocardial structural and functional changes in patients with liver cirrhosis awaiting liver transplantation: A comprehensive cardiovascular magnetic resonance and echocardiographic study. [21]
Le et al.	**The application of exercise stress cardiovascular magnetic resonance in patients with suspected dilated cardiomyopathy. **[22]
Qi et al.	**Respiratory motion-compensated high-resolution 3D whole-heart t1p mapping.** [23]
Holtstiege et al.	Clinical experience regarding safety and diagnostic value of cardiovascular magnetic resonance in patients with a subcutaneous implanted cardioverter/defibrillator (S-ICD) at 1.5 T. [24]
Snel et al.	Cardiovascular magnetic resonance native T2 and T2* quantitative values for cardiomyopathies and heart transplantations: a systematic review and meta-analysis. [4, 5]
Podlesnikar et al.	Left ventricular functional recovery of infarcted and remote myocardium after ST-Segment elevation myocardial infarction (METOCARD-CNIC randomized clinical trial substudy. [25]
Backhaus et al.	Real-time cardiovascular magnetic resonance T1 and extracellular volume fraction mapping for tissue characterisation in aortic stenosis. [26]
Le et al.	**Sub-segmental quantification of single (stress)-pass perfusion CMR improves the diagnostic accuracy for detection of obstructive coronary artery disease**. [27]
Domenech-Ximenos et al.	**Prevalence and pattern of myocardial late enhancement in cardiac magnetic resonance of highly trained endurance athlete.** [28]
Xu et al.	Multiparametric cardiovascular magnetic resonance characteristics and dynamic changes in myocardial and skeletal muscles in idiopathic inflammatory cardiomyopathy. [29]
Kato et al.	Incremental prognostic value of coronary flow reserve determined by phase-contrast cine cardiovascular magnetic resonance of the coronary sinus in patients with diabetes mellitus. [30]
Liu et al.	Myocardial fibrosis in asymptomatic and symptomatic severe chronic primary mitral regurgitation and relationship to tissue characterization and LV function on cardiovascular magnetic resonance. [31]
Kocaoglu et al.	**Breath-hold and free-breathing quantitative assessment of biventricular volume and function using compressed SENSE: a clinical validation in children and young adults**. [32]
Craven et al.	**Exercise cardiovascular magnetic resonance: development, current utility and future applications.** [3]
Correia et al.	**Accelerated high-resolution free-breathing 3D whole-heart T2-prepared black-blood and bright-blood cardiovascular magnetic resonance**. [33]
Xu et al.	Layer-specific strain in patients with heart failure using cardiovascular magnetic resonance: not all layers are the same. [32]
Pennig et al.	Clinical application of free-breathing 3D whole heart late gadolinium enhancement cardiovascular magnetic resonance with high isotropic spatial resolution using Compressed SENSE. [35]

Bold manuscripts were also selected for 2020 *JCMR* Journal Club presentation

## Social media

I am very much a social media novice, but the *JCMR* continues to be very active on Twitter with the handle “Journal of CMR.” Tweets go out with the publication of each manuscript publication and announcing each Journal Club. This activity is coordinated by our two Social Media editors, Drs. Juan Lopez-Mattei and Purvi Parwani. According to Dr. Parwani and Lopez-Mattei, as of 12/21/2021, we had 3830 followers (a 20% increase over last year). For comparison, the *Journal of the American Society of Echocardiography* (*JASE*) has 3301 followers, the *Journal of Cardiac Computed Tomography* (*JCCT*) has 3226 followers, Circulation: Cardiovascular Imaging has 2603 followers, and the *Journal of Nuclear Cardiology* has 1480 followers. We thank our Twitter audience for the strong #whyCMR following. 

## Gerald M. Pohost and Dudley Pennell Awards

In recognition of the efforts of our inaugural editor-in-chief, Dr. Gerald M. Pohost, (Fig. 2) for the past 14 years, the *JCMR* has awarded the Pohost Prize to that manuscript deemed by the associate editors and editorial board to be the best/most important manuscript published in the prior year. The associate editors and I select the Pohost finalists (Table 7) and the entire editorial board votes on the top prize. At the virtual 2021 SCMR Scientific Sessions annual meeting, the 14th Gerald M. Pohost Prize was awarded to Dr. Bandettini (Fig. 3) and co-workers for their manuscript” A comparison of cine CMR imaging at 0.55 T and 1.5 T” [15]. The Pohost Runner-up Prize was awarded to Dr. Reddy (Fig. 3) and colleagues for their publication, “Invasive cardiovascular magnetic resonance (iCMR) for diagnostic right and left heart catheterization using an MR conditional guidewire and passive visualization in congenital heart disease” [16].

**Table 7:** 2021 Gerald M. Pohost Award Finalists. Dr. Bandettini was the recipient of the 14th Gerald M. Pohost Award. Dr. Reddy was the runner-up

Bandettini et al.	A comparison of cine CMR imaging at 0.55 T and 1.5 T. [15]
Champ-Rigot et al.	Clinical outcomes after primary prevention defibrillator implantation are better predicted when the left ventricular ejection fraction is assessed by cardiovascular magnetic resonance. [36]
Jacobs et al.	Direct measurement of atrioventricular valve regurgitant jets using 4D flow cardiovascular magnetic resonance is accurate and reliable for children with congenital heart disease: a retrospective cohort study. [37]
Kranzusch et al.	Z-score mapping for standardized analysis and reporting of cardiovascular magnetic resonance modified Look-Locker inversion recovery (MOLLI) T1 data: Normal behavior and validation in patients with amyloidosis. [38]
Lindemann et al.	Clinical utility of cardiovascular magnetic resonance imaging in patients with implantable cardioverter defibrillators presenting with electrical instability or worsening heart failure symptoms [39]
Liu et al.	Myocardial fibrosis in asymptomatic and symptomatic chronic severe primary mitral regurgitation and relationship to tissue characterisation and left ventricular function on cardiovascular magnetic resonance. [31]
Martini et al.	Deep learning to diagnose cardiac amyloidosis from cardiovascular magnetic resonance. [40]
Olivieri et al.	Normal right and left ventricular volumes prospectively obtained from cardiovascular magnetic resonance in awake, healthy, 0–12 year old children. [41]
Qiao et al.	Quantitative evaluation of carotid atherosclerotic vulnerable plaques using in vivo T1 mapping cardiovascular magnetic resonaonce: validation by histology. [42]
Reddy et al.	Invasive cardiovascular magnetic resonance (iCMR) for diagnostic right and left heart catheterization using an MR-conditional guidewire and passive visualization in congenital heart disease. [16]

At that meeting, we also presented the 3rd Pennell Award in recognition of the foresight of *JCMR*’s 2nd Editor-in-Chief, Professor Dudley J. Pennell (Fig. 2) to transition the *JCMR* to the open-access platform (a decision (spearheaded by then SCMR Publications Committee chairman, Dr. Matthias Friedrich). Their decision markedly improved *JCMR*’s visibility and impact factor. The Pennell award is for that original manuscript that has most contributed to the *Journal’s* impact factor for the calendar year 3 years prior to the award. The 3rd Dudley J. Pennell Prize was awarded to Dr. Petersen (Fig. 3) et al. for their publication, “Reference ranges for cardiac structure and function using cardiovascular magnetic resonance (CMR) in Caucasians from the UK Biobank population cohort” [17] with the runner-up Pennell Award was given to Dr. Peter Kellman (Fig. 3) and colleagues for publication, “Myocardial perfusion cardiovascular magnetic resonance: optimized dual sequence and reconstruction for quantification” [18].

Stay tuned for the 15th Pohost and 4th Pennell Awards that will presented at the 23nd Scientific Sessions of the *Society* this February!

## Tribute to Nathaniel Reichek

**Fig. 4:**
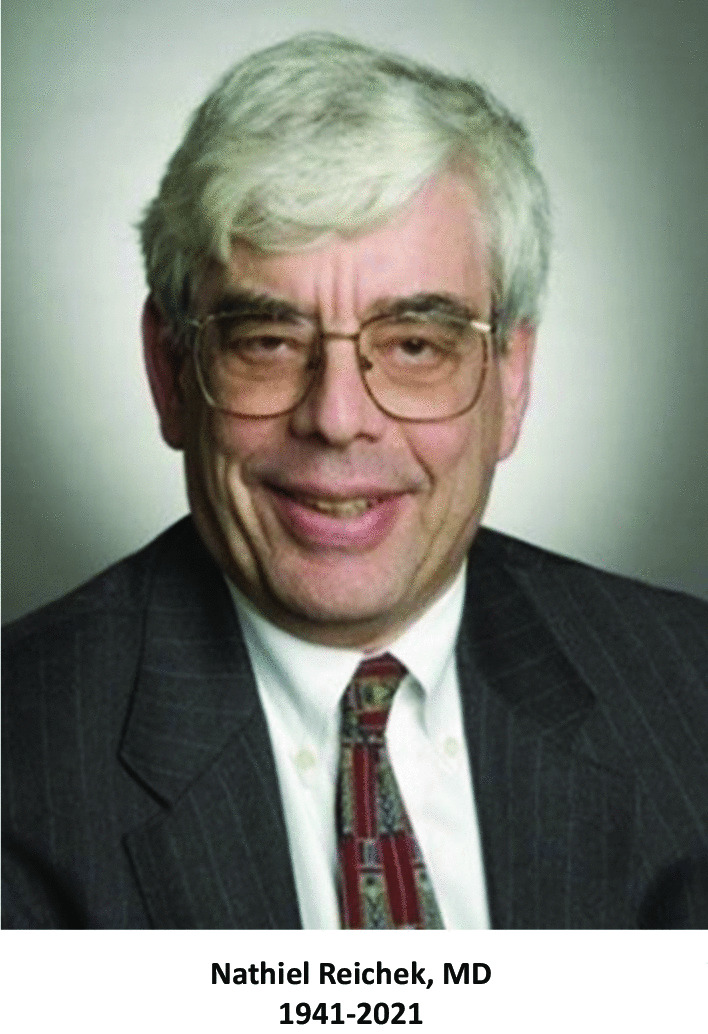
Dr. Nathaniel Reichek (1941–2021). SCMR founding member, former president, Gold Medal recipient, *JCMR* senior advisor and frequent guest editor

This year the SCMR and the greater CMR community lost one of our founding fathers. Dr. Nathaniel Reichek (Fig. 4), a true giant in our field. Nat was literally “in the room” when the SCMR was founded, served as our 3rd president, was a 2017 recipient of the SCMR Gold Medal, and for many years spearheaded the United States CMR Advocacy Committee. He was a very strong advocate and tireless worker for CMR, for the SCMR, and for the *JCMR.* I had the great personal privilege of knowing Nat for over 3 decades and will cherish our many discussions over the years. For my tenure as editor-in-chief, Nat was my “go to” person for conflict-of-interest manuscripts. His command of CMR was almost unparalleled, and he readily gave his time to help the Journal and all who inquired of his opinion. While we didn’t agree on every issue, Nat was a gentleman of high integrity, and I miss him at multiple levels. May his memory be for a blessing.

## Manuscripts—WordCloud

**Fig. 5:**
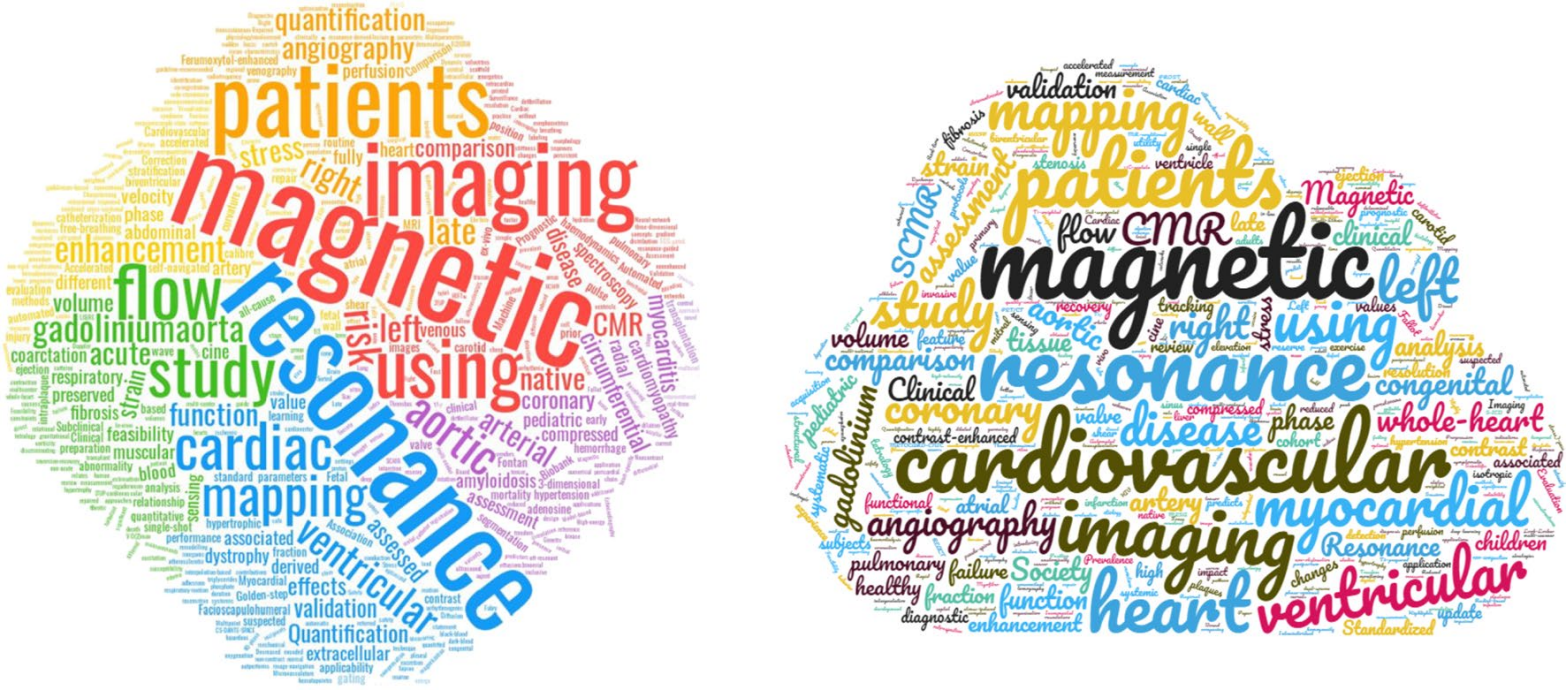
WordCloud of **A** 2019 and **B** 2020 *JCMR* manuscript titles

As in last year’s review, I again chose to create a Wordcloud (https://www.wordclouds.com) of the 2019 and 2020 *JCMR* titles (Fig. 5). As in 2019, the most common JCMR manuscript title words were magnetic, cardiovascular, resonance with 2020 followed by imaging, heart, ventricular and myocardial.

I hope you have found the annual “State of our *JCMR*” informative. I remain your captain until December 2022, but as members of the *SCMR*, it is really your Journal for which I thank you for allowing me to provide stewardship. I close by again thanking the entire *JCMR* team and you, our readership. We will continue our efforts to maintain the average time-to-first decision to < 35 days in 2022 and hope to be able to include anonymized reviewer comments with each manuscript. Remember to also join us for our monthly *JCMR* Journal Club for which I hope CME for attendance will be available later this year!

Wishing you a happy, healthy, and safe 2022. I am confident the brilliant scientific minds of our world will successfully get us through the COVID-19 pandemic, but like other disasters, we are unlikely to return to our pre-Covid state. Stay safe, advance the field of CMR, and take a moment to make sure that every day is special.

## Data Availability

Data sharing not applicable to this article as no datasets were generated or analyzed.

## Abbreviations

APC: Article processing charge
CME: Continuing medical education
JCMR: Journal of Cardiovascular Magnetic Resonance
SCMR: Society for Cardiovascular Magnetic Resonance
